# Investigation of the sclerosing effect of increasing doses of polidocanol on pulmonary vessels: Experimental study

**DOI:** 10.1097/MD.0000000000041270

**Published:** 2025-01-17

**Authors:** Duygu Zorlu, Imran Özdemir, Nuray Bayar Muluk, Enes Güngör, Evrim Yilmaz, Cemal Cingi

**Affiliations:** a Department of Pulmonology, Medicana İnternational İzmir Hospital, Izmir, Türkiye; b Department of Pulmonology, Turkiye Gazetesi Private Hospital, Istanbul, Türkiye; c Department of Otorhinolaryngology, Kirikkale University, Kirikkale, Türkiye; d Department of Otorhinolaryngology, Eshisehir Osmangazi University, Eskisehir, Türkiye; e Department of Pathology, Eshisehir Osmangazi University, Eskisehir, Türkiye.

**Keywords:** polidocanol, pulmonary vessels, sclerotherapy

## Abstract

**Background:**

Percutaneous sclerotherapy as endovascular treatment may cause severe complications beside the target area. Pulmonary embolism and thrombosis may occur. In this regard, our study aimed to reveal whether increasing systemic doses of polidocanol affects the coronary or pulmonary alveolar levels.

**Methods:**

Twenty-one Albino New Zealand rabbits, 7 rabbits per group, were used. The first group served as a control and received a 0.5 mL injection of saline; the second group received a single injection of 0.5 mL 1% polidocanol; and the third group received a single injection of 0.5 mL 2% polidocanol via the femoral vein. After anesthesia-induced decapitation on day 3, the experimental rabbits’ lungs were removed in their entirety and sent to pathology for analysis. Micron-thick slices.

**Results:**

In all lung specimens of the first 2 groups, no narrowing or obstruction was detected in the pulmonary vessels in macroscopic and microscopic evaluation. No emphysematous changes or congestion were observed. In the third group’s animal lungs, congestion in pulmonary vessels, emphysematous changes, and intra-alveolar hemorrhage were found.

**Conclusion:**

In our study, a single injection of 0.5 mL 2% polidocanol via the femoral vein. Animal lungs, congestion in pulmonary vessels, emphysematous changes, and intra-alveolar hemorrhage were found. As a result, our study demonstrated pulmonary vascular damage with increasing doses of polidocanol. So, the dose of polidocanol should be used in a limited manner.

## 1. Introduction

Vascular malformations are treated using sclerotherapy agents and embolization treatments such as alcohol, polidocanol, or sodium tetradecyl sulfate foam. Percutaneous sclerotherapy is provided with polidocanol to treat venous malformations.^[1–3]^ Apart from local and allergic side effects, almost no significant side effects have been reported that are directly associated with polidocanol. These endovascular treatments also have the possibility of serious complications such as pulmonary embolism and thrombosis.^[4,5]^

The endothelium and the entire vascular wall are susceptible to significant damage from various sclerosants. Sclerosis represents a prolonged outcome of successful sclerotherapy, which converts veins into fibrous cords. The objective of sclerotherapy extends beyond merely inducing thrombosis, which may result in recanalization; it aims to transform the vessel into a fibrous cord permanently. Since this chord cannot be recanalized, the functional outcome parallels a varicose vein removal procedure.^[3,6]^

Polidocanol is the preferred medication for sclerotherapy. The plasticizing, emollient, and surfactant properties have contributed to its extensive application. Concerns regarding its security and reliability are increasing. Polidocanol functions as a sclerosant intended for administration to endothelial cells. Endothelial cells participate in various physiological functions. Angiogenesis, immunological responses, coagulation, tissue permeability, vascular tone, and vessel repair are all included in this category.^[7–9]^

However, there are limited studies on the effects of an agent, a sclerosant whose mechanism of action proceeds through a cascade involving the shaped elements of the blood, on the significant organs it will encounter first if it enters the systemic circulation.^[1–3]^ In this regard, our study aimed to reveal whether increasing systemic doses of polidocanol have an effect at the coronary or pulmonary (alveolar) level.

## 2. Materials and methods

Twenty-one Albino New Zealand rabbits weighing 2500 and 3000 grams were used. Following 24 hours of acclimation, the rabbits were randomly assigned to 3 groups, with 7 rabbits per group.

The usual dose of polidocanol injection is 0.5%. In this study, we aimed to try higher doses. The first group served as a control and received a 0.5 mL injection of saline; the second group received a single injection of 0.5 mL 1% polidocanol (a high dose for clinical usage); and the third group received a single injection of 0.5 mL 2% polidocanol (a double dose for clinical usage) via the femoral vein.

**Figure 1.**
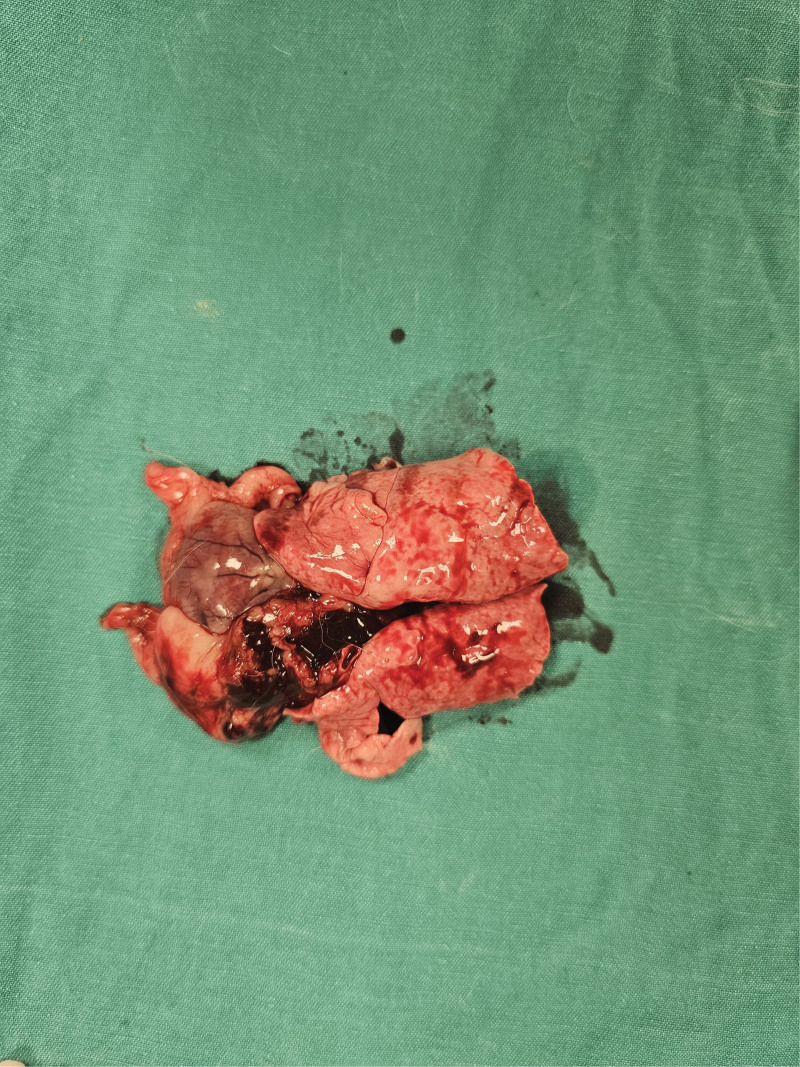
Lungs extracted after the experiment before sending to pathological examination.

All experimental animals were sacrificed at the end of the study with a lethal dose of thiopental sodium (150 mg/kg). The rabbits’ lungs were dissected and sent to pathology for analysis (Fig. 1).

Lung specimens were processed at numbers 1 to 21. After pathological tissue tracking, Haematoxylin and Eosin staining was performed on 4-micron-thick slices. The pathologist performing the lung tissue analysis was blinded to the treatment groups.

The study was conducted in accordance with the Basic & Clinical Pharmacology & Toxicology policy for experimental and clinical studies.^[4]^

## 3. Results

In all lung specimens of the first 2 groups, macroscopic and microscopic evaluation detected no narrowing or obstruction of the pulmonary vessels. No emphysematous changes or congestion were observed.

**Figure 2.**
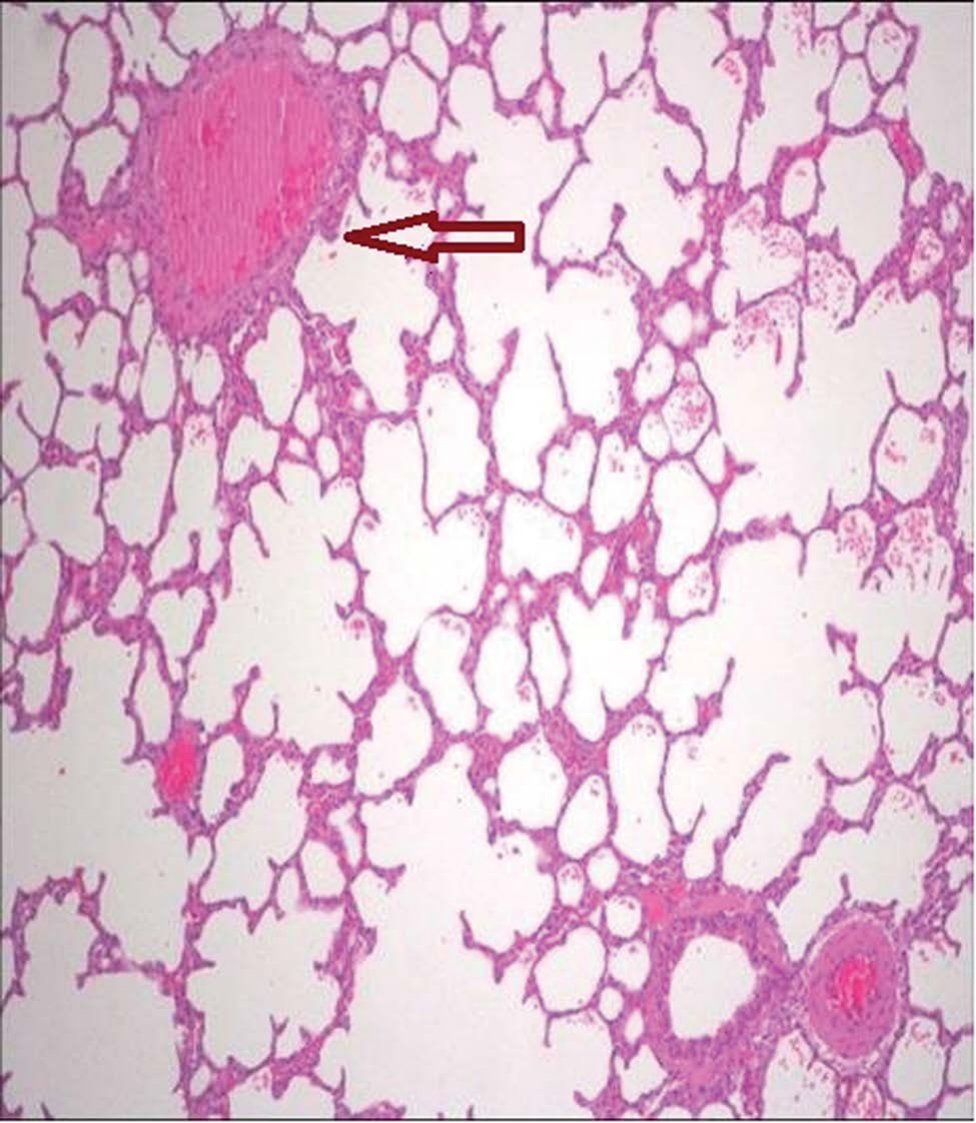
Congestion (excessive accumulation of blood within a vessel) in pulmonary vessels (arrows indicate pathological areas) (H&E, ×10). H&E = Haematoxylin and Eosin.

**Figure 3.**
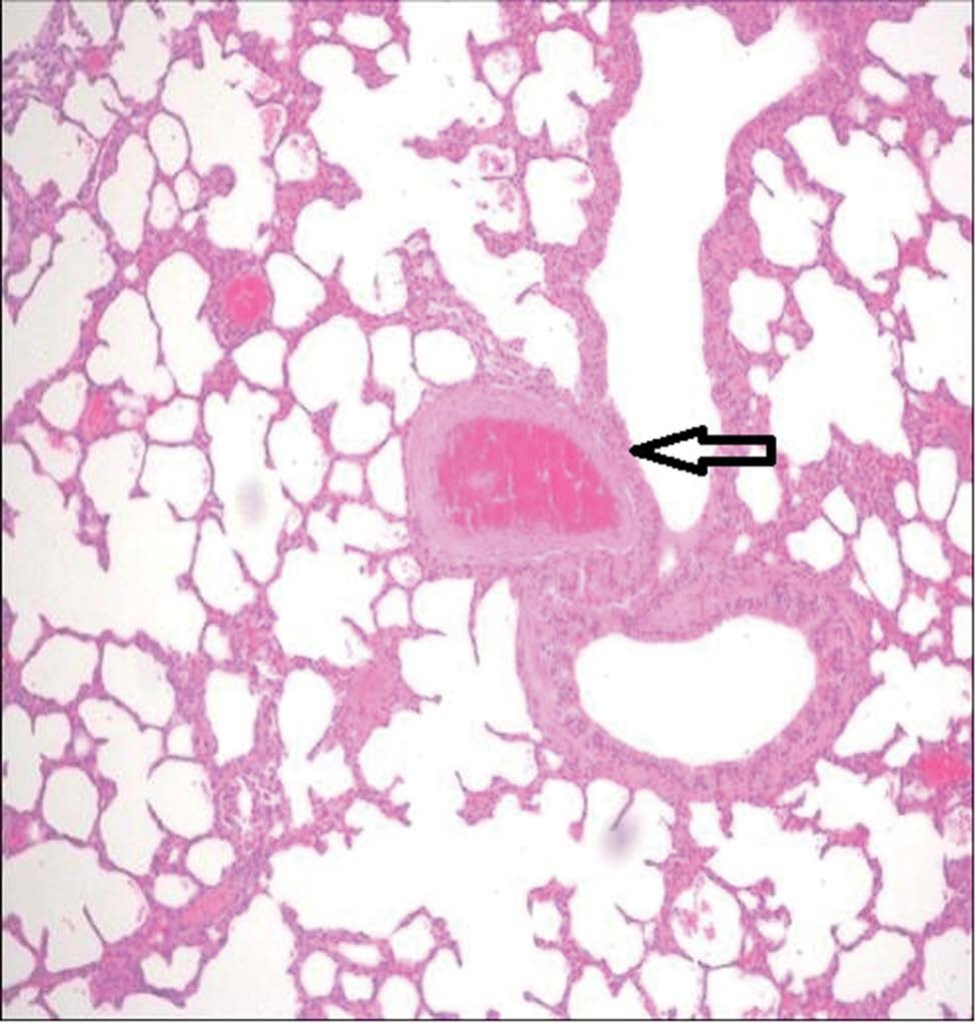
Congestion (excessive accumulation of blood within a vessel) in pulmonary vessels (arrows indicate pathological areas) (H&E, ×10). H&E = Haematoxylin and Eosin.

**Figure 4.**
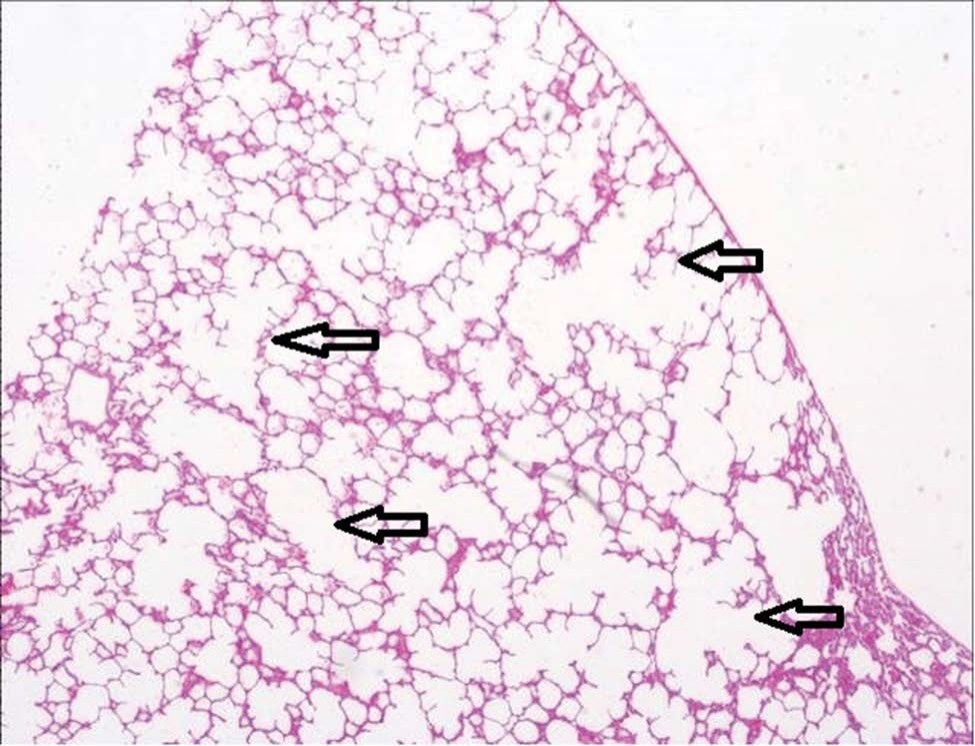
Emphysematous changes (airspace enlargement) in the lung parenchyma (arrows indicate pathological areas) (H&E, ×4). H&E = Haematoxylin and Eosin.

**Figure 5.**
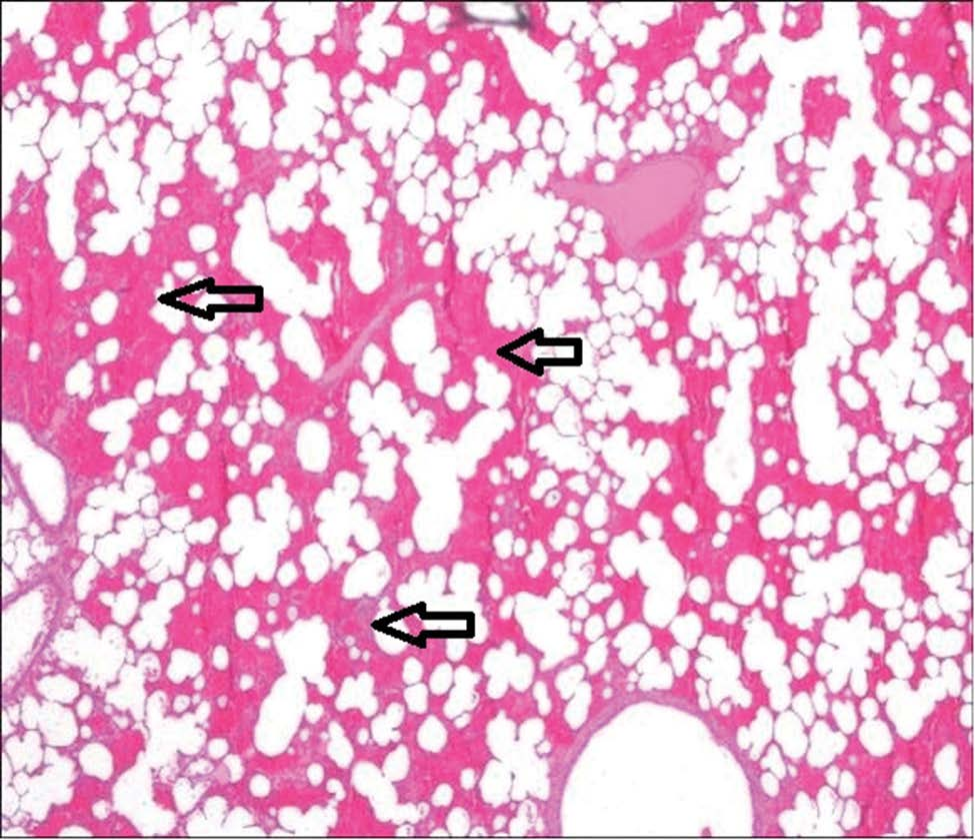
Areas of intra-alveolar hemorrhage (abundant red blood cells in the airspaces) (arrows indicate pathological areas) (H&E, ×4). H&E = Haematoxylin and Eosin.

The third group’s animal lungs showed congestion in pulmonary vessels, emphysematous changes, and intra-alveolar hemorrhage (Figs. 2–5).

## 4. Discussion

The effectiveness and safety of foam injection sclerotherapy as a minimally invasive treatment for varicose veins have been documented in large case series.^[5–7]^ Studies have been conducted on the effectiveness of the treatment, but reports on side effects, especially thrombosis and pulmonary complications, are rare. In our research, thrombosis and pulmonary complications were evaluated with animal experiments at increasing doses of polidocanol – because the effectiveness of the treatment increases. Our study found a single injection of 0.5 mL 2% polidocanol via the femoral vein, animal lungs, congestion in pulmonary vessels, emphysematous changes, and intra-alveolar hemorrhage.

Sclerotherapy addresses venous abnormalities by sclerosing subfascial veins and removing intracutaneous, subcutaneous, and trans-fascial varicose veins. This is the method for the aesthetic treatment and prevention of varicose veins. The endothelium and the entire arterial wall are susceptible to significant damage from several sclerosants. Sclerosis is a prolonged consequence of successful sclerotherapy, which entails converting veins into fibrous cords.^[8,9]^ The objective of sclerotherapy is to permanently transform the vessel into a fibrous cord rather than merely inducing thrombosis, which may result in recanalization. Since this chord cannot be recanalized, the functional outcome resembles a varicose vein excision treatment.

Polidocanol is the preferred drug for sclerotherapy. Its plasticizing, emollient, and surfactant characteristics have resulted in its extensive application. Nonetheless, apprehensions regarding its security and dependability are increasing. Polidocanol is a sclerosant intended for administration to endothelial cells. Endothelial cells participate in a wide array of physiological processes.^[10–12]^ This category includes angiogenesis, immune responses, coagulation, tissue permeability, vascular tone, and vessel repair.

Studies on polidocanol and thrombosis are limited. Neurological complications such as transient visual impairment and transient confusional state have been described in a single randomized controlled study but are rare.^[6]^ Seven events were reported in 2453 patients, but the incidence was attributed to the foam production method. In another series, including 12,500 patients, 4 cases of transient scotoma with or without migraine were reported.^[3]^ A case report has been published documenting paralysis in a patient with a patent foramen ovale, but this occurred 3 days after varicose vein sclerotherapy, and the relationship was interpreted as tenuous at best.^[6]^ Lung symptoms such as cough have also been reported.^[13]^

In a study using polidocanol foam (Tessari method) to analyze venous thromboembolism (VTE) prevalence after ultrasound-guided foam sclerotherapy, the primary outcome was VTE prevalence, and the secondary outcome was possible risk factors. It was observed that VTE occurred at a rate of 0.49% (pulmonary embolism 0.3%) in an average of 44.0 ± 42.2 days. Male gender, personal or family history of phlebitis or DVT, and high-caliber varicose veins were significantly associated with VTE. It has been determined that a low incidence of VTE, male gender, a personal or family history of VTE, and a varicose vein diameter larger than 7 mm increase the risk.^[14]^

Another study prospectively evaluated the clinical response of low-flow vascular malformations treated with 1% polidocanol foam sclerotherapy, assuming that large-scale lesions reduce the effectiveness and satisfaction of sclerotherapy. It has been stated that treatment of low-flow vascular malformations with 1% polidocanol foam is safe and effective and improves symptoms and quality of life. It has been determined that treatment failure increases in large-diameter lesions (>10 cm). Patients reported recurrent symptoms; other methods were needed for successful treatment in this group.^[15]^ Our study revealed that higher doses of polidocanol can be given to these patients, but this may cause more significant venous vascular complications.

In another report, fatal pulmonary embolism was reported after sclerotherapy, and it was stated that the effectiveness of treatment increased with increasing doses, but complications may increase.^[16]^

The optimal volume of foam to treat trunk varicose veins is controversial. A recent European consensus statement recommended 6 to 8 mL per session, but published reports have used 3 to 30 mL.^[7]^ Air embolism can be fatal when a volume > 1 mL/kg enters the venous system but can cause problems with a volume as small as 50 mL. It has been interpreted that larger volumes of foam are associated with a higher incidence of deep vein thrombosis.^[7]^

No embolic complications have been reported with other nonoperative methods, such as endovenous laser and radiofrequency ablation (VNUS Medical Technologies, San Jose, CA).^[17]^ Complication rates are lower than open surgery, but 1 study suggested that the rate of deep vein thrombosis is higher with radiofrequency ablation.^[18,19]^

In conclusion, limited studies exist on thrombosis and pulmonary side effects in treatment with polidocanol, and our study demonstrated pulmonary vascular damage with increasing doses of polidocanol. The risk of developing pulmonary embolism increases in patients with atherosclerosis, obesity, oral contraceptive use, female gender, and β-blocker use. Therefore, in cases where sclerotherapy will be applied with polidocanol, these situations should be considered when determining the indication, and the dose of polidocanol should be used in a limited manner.

## 5. Conclusion

Polidocanol sclerotherapy aims to treat varicose veins locally. However, at high enough concentrations, polidocanol may permeate all circular networks and impact tissues other than our primary target. We thought the buildup of plaque in the pulmonary arteries was the initial stage of their sclerosis. To mitigate the risk of pulmonary complications, using the lowest effective dosage in local sclerosis application will be helpful.

## Author contributions

**Conceptualization:** Duygu Zorlu, Imran Özdemir, Nuray Bayar Muluk, Cemal Cingi.

**Data curation:** Duygu Zorlu, Imran Özdemir, Enes Güngör, Evrim Yilmaz, Cemal Cingi.

**Formal analysis:** Duygu Zorlu, Imran Özdemir, Nuray Bayar Muluk, Evrim Yilmaz, Cemal Cingi.

**Funding acquisition:** Duygu Zorlu.

**Investigation:** Duygu Zorlu, Imran Özdemir, Nuray Bayar Muluk, Enes Güngör, Evrim Yilmaz, Cemal Cingi.

**Methodology:** Duygu Zorlu, Imran Özdemir, Nuray Bayar Muluk, Enes Güngör, Evrim Yilmaz, Cemal Cingi.

**Project administration:** Duygu Zorlu, Imran Özdemir, Nuray Bayar Muluk, Enes Güngör, Evrim Yilmaz, Cemal Cingi.

**Resources:** Duygu Zorlu, Imran Özdemir, Enes Güngör.

**Software:** Duygu Zorlu, Imran Özdemir, Nuray Bayar Muluk, Cemal Cingi.

**Supervision:** Duygu Zorlu, Imran Özdemir, Nuray Bayar Muluk, Enes Güngör, Evrim Yilmaz, Cemal Cingi.

**Validation:** Duygu Zorlu, Imran Özdemir, Nuray Bayar Muluk, Enes Güngör, Evrim Yilmaz, Cemal Cingi.

**Visualization:** Duygu Zorlu, Imran Özdemir, Evrim Yilmaz, Cemal Cingi.

**Writing – original draft:** Duygu Zorlu, Imran Özdemir, Enes Güngör, Evrim Yilmaz.

**Writing – review & editing:** Duygu Zorlu, Imran Özdemir, Nuray Bayar Muluk, Evrim Yilmaz, Cemal Cingi.
